# A pilot study to compare the detection of HPV-16 biomarkers in salivary oral rinses with tumour p16^INK4a^ expression in head and neck squamous cell carcinoma patients

**DOI:** 10.1186/s12885-016-2217-1

**Published:** 2016-03-03

**Authors:** Ryan C. Chai, Yenkai Lim, Ian H. Frazer, Yunxia Wan, Christopher Perry, Lee Jones, Duncan Lambie, Chamindie Punyadeera

**Affiliations:** The University of Queensland Diamantina Institute, The University of Queensland, Translational Research Institute, 37 Kent St, Woolloongabba, QLD Australia; Present address: Garvan Institute of Medical Research, 384 Victoria St, Darlinghurst, NSW Australia; The School of Biomedical Sciences, Institute of Health and Biomedical Innovations, Queensland University of Technology, 60 Musk Avenue, Kelvin Grove, QLD Australia; Princess Alexandra Hospital, 199 Ipswich Rd, Woolloongabba, QLD Australia; IQ Pathology, 6/11 Donkin St, West End, QLD Australia

**Keywords:** HPV, HNSCC, OPSCC, Saliva, Early detection

## Abstract

**Background:**

Human papilloma virus-16 (HPV-16) infection is a major risk factor for a subset of head and neck squamous cell carcinoma (HNSCC), in particular oropharyngeal squamous cell carcinoma (OPSCC). Current techniques for assessing the HPV-16 status in HNSCC include the detection of HPV-16 DNA and p16^INK4a^ expression in tumor tissues. When tumors originate from hidden anatomical sites, this method can be challenging. A non-invasive and cost-effective alternative to biopsy is therefore desirable for HPV-16 detection especially within a community setting to screen at-risk individuals.

**Methods:**

The present study compared detection of HPV-16 DNA and RNA in salivary oral rinses with tumor p16^INK4a^ status, in 82 HNSCC patients using end-point and quantitative polymerase chain reaction (PCR).

**Results:**

Of 42 patients with p16^INK4a^-positive tumours, 39 (sensitivity = 92.9 %, PPV = 100 % and NPV = 93 %) had oral rinse samples with detectable HPV-16 DNA, using end-point and quantitative PCR. No HPV-16 DNA was detected in oral rinse samples from 40 patients with p16^INK4a^ negative tumours, yielding a test specificity of 100 %. For patients with p16^INK4a^ positive tumours, HPV-16 mRNA was detected using end-point reverse transcription PCR (RT-PCR) in 24/40 (sensitivity = 60 %, PPV = 100 % and NPV = 71 %), and using quantitative RT-PCR in 22/40 (sensitivity = 55 %, PPV = 100 % and NPV = 69 %). No HPV-16 mRNA was detected in oral rinse samples from the p16^INK4a^-negative patients, yielding a specificity of 100 %.

**Conclusions:**

We demonstrate that the detection of HPV-16 DNA in salivary oral rinse is indicative of HPV status in HNSCC patients and can potentially be used as a diagnostic tool in addition to the current methods.

**Electronic supplementary material:**

The online version of this article (doi:10.1186/s12885-016-2217-1) contains supplementary material, which is available to authorized users.

## Background

Oral squamous cell carcinoma (OSCC) and oropharyngeal squamous cell carcinoma (OPSCC) are the most common types of head and neck squamous cell carcinoma (HNSCC), accounting for 263,900 new cases and 128,000 deaths worldwide [1]. These cancers are highly curable if detected early and the most common treatments include surgery, radiation therapy, chemotherapy or a combination of these three treatments. Tobacco smoking and alcohol consumption are major risk factors for HNSCC, with approximately 80 % of cases attributed to tobacco exposure [2]. Alcohol consumption can act synergistically with tobacco to increase the risk of HNSCC [3].

In recent decades, the overall incidence of HNSCC is in the decline in the developed world due to a reduction in the consumption of tobacco. However, there is a concomitant increase in the incidence of OPSCC attributable to human papilloma virus (HPV) infection, in particular the high risk HPV (HR-HPV) strain HPV-16 [4]. About 40–80 % of OPSCC cases in the USA are caused by HPV, whereas in Europe the percentage varies from about 90 % in Sweden to less than 20 % in countries with the highest rates of tobacco use [4]. Unlike tobacco-related HNSCC, patients with HPV-positive OPSCC are usually less likely to have any history of excess tobacco or alcohol consumption. Furthermore, it is estimated that tumors in the oropharynx are five times more likely to be HPV-positive than those in the oral cavity, larynx or hypopharynx [5].

HPV-positive OPSCC has genetic alterations that are a direct result of HPV oncoproteins, E6 and E7, which inactivate the tumor suppressor gene products, p53 and Rb respectively. During immortalization of host cells, the E7 protein of HR-HPV binds to Rb, resulting in the compensatory over-expression of the tumor suppressor gene p16^INK4A^ in HPV-infected tumor cells [6]. The immunohistochemical (IHC) analysis of p16^INK4A^ in HNSCC tumor biopsies has been shown to serve as a surrogate marker to identify HPV infection in tumor. Tests that measure HPV DNA or RNA directly in tumor samples have also been reported recently, using in situ hybridization (ISH) or PCR for detection of HPV DNA, and RT-PCR and RNA ISH for HPV-related RNA [5]. However, current methods for the detection of HPV in OPSCC patients require tumor biopsy samples, often from challenging anatomical sites such as the tonsillar crypts, which may hamper early detection.

Oral fluids have been shown to contain different analytes such as hormones, steroids, antibodies, growth factors, cytokines, chemokines and drugs, which may reflect local and systemic disease states [7–10]. They also contain whole cells, genetic materials and proteins that may reflect cellular alterations in pathogen-infected cells [11]. We therefore hypothesized that oral fluids could serve as a non-invasive and cost-effective alternative to biopsy for the detection of HPV-16, as well as potentially allowing early cancer detection. The current study evaluates the feasibility of using HPV-16 DNA and RNA in oral fluids as biomarkers for p16 ^INK4a^ - positive HNSCC.

## Methods

### Study patients

Subjects in the current study were recruited from patients treated in the Ear, Nose and Throat (ENT) Department of the Princess Alexandra Hospital in Woolloongabba, Queensland, Australia between 2013 and 2015. This study was approved by the University of Queensland Medical Ethical Institutional Board (HREC: 2014000679), Queensland University of Technology Medical Ethical Review Board (HREC: 1400000616) and by the Princess Alexandra Hospital Ethics Review Board (HREC: HREC/12/QPAH/381). All patients with a primary cancer of the oral cavity and oropharynx or patients with loco-regional metastasis with oral/oropharyngeal origin who agreed to sign the information and consent form were enrolled. Demographic data and risk factors associated with oral and oropharyngeal cancers were collected by a patient questionnaire. A pathology report was included for each patient, which contained pathological staging of the tumor, histopathological classification and HPV status based on p16^INK4a^ immunohistochemistry (IHC). p16^INK4a^ IHC was performed at the pathology laboratory of the Princess Alexandra Hospital, Woolloongabba, Australia using CINtec Histology Kit (Roche MTM Laboratories, Heidelberg, Germany) according to manufacturer’s instructions. p16 ^INK4a^ status of tumor sections was assessed and established by qualified pathologists unaware of the HPV oral rinse results.

### Oral rinse collection and processing

Oral rinse samples were collected by having the patients swish and gargle for 1 min with 10 mL 0.9 % saline. Samples were immediately frozen on dry ice and transported to the laboratory for processing. Samples were then thawed, centrifuged at 1000 × *g* for 10 mins at 4 °C. Cell pellets were resuspended in sterile PBS for DNA extraction or Qiazol (Qiagen, Valencia, CA, USA) for RNA extraction and stored at−80 °C until further processing.

### DNA and RNA extraction from oral rinses

Oral exfoliated cell pellets were resuspended in sterile PBS and DNA was extracted using the QIAmp DNA Mini Kit (Qiagen) according to the manufacturer’s instructions. Total RNA was extracted from oral exfoliated cell pellet resuspended in Qiazol as described previously [12]. Briefly, 200 μL of chloroform was added to 800 μL of QIAzol containing oral exfoliated cells and vortexed for 10 min. The sample was then centrifuged at 10,000 × *g* for 10 min at 4 °C and the aqueous phase was collected. Chloroform (200 μL) was added to the aqueous phase, vortexed for 5 min followed by centrifugation at 10,000 × *g* for 10 min at 4 °C. The aqueous phase was collected and an equal volume of isopropanol was added for RNA precipitation overnight at −20 °C. RNA was pelleted by centrifugation at 10,000 × *g* at 4 °C for 20 min, washed with 1 mL of 70 % ethanol and centrifuged again at 10,000 × *g* for 5 min at 4 °C. Supernatant was removed and the samples were air dried for at least 30 min. The RNA pellet was re-suspended in 15 μL RNase- free water. DNA and RNA samples were assessed for purity and quantified on a Nanodrop 1000 Spectrophotometer (Thermo Fisher Scientific, Pittsburgh, PA, USA).

### HPV-16 DNA detection with end-point PCR in oral rinse samples

For the detection of HPV-16 DNA in oral rinse samples, we used end-point PCR method as well as quantitative PCR (qPCR). Specific primers were used for the amplification of a region spanning the E6 and E7 genes of the HPV-16 genome [13] and primers for a housekeeping gene (β-globin) [14] was run in parallel to normalize the amount of DNA input (Table 1A). The PCR reaction mix consisted of 50 ng of DNA isolated from oral rinse, 1 μM of each primer, 1x Emerald AMP MAX HS PCR mastermix (Takara Bio, Otsu, Shiga, Japan) in a total volume of 12.5 μL. PCR reaction condition consisted of an initial denaturation at 95 °C for 2 min followed by 40 cycles of; 95 °C for 30 s, annealing for 30 s at 62 °C for HPV-16 E6/E7 or 60 °C for β-Globin, and extension at 72 °C for 30 s. A final extension at 72 °C before cooling to 4 °C was performed. The PCR products were subjected to gel electrophoresis.

**Table 1:** Sequences of polymerase chain reaction primers and probes for HPV-16 specific DNA and transcript

A. End-point PCR primers for the detection and amplification of HPV-16 specific DNA HPV-16 E6/E7 forward primer: 5’ -CCCAGCTGTAATCATGCATGGAGA-3’ reverse primer: 5’ -GTGTGCCCATTAACAGGTCTTCCA-3’
β-globin forward primer: 5’ -CAACTTCCACGGTTCACC-3’ reverse primer: 5’ -GAAGAGCCAAGGACAGGTAC-3’
B. Quantitative PCR primers for the detection and amplification of HPV-16 specific DNA HPV-16 E7 forward primer: 5’ -GATGAAATAGATGGTCCAGC-3’ reverse primer: 5’ -GCTTTGTACGCACAACCGAAGC-3’
C. End-point RT-PCR primers for the detection and amplification of HPV-16 specific transcript HPV-16 E6 forward primer: 5’ -CAGGAGCGACCCAGAAAGTT-3’ reverse primer: 5’ -GCAGTAACTGTTGCTTGCAGT-3’ GAPDH forward primer: 5’ -TTGCCCTCAACGACCACTTT-3’ reverse primer: 5’ -TTGCCCTCAACGACCACTTT-3’
D. Taqman probes for the detection and amplification of HPV-16 specific transcript HPV-16 E6/E7 forward primer: 5’ -(MGB)-CCAGCTGTAATCATGCATGGA-3’ reverse primer: 5’ -(MGB)-CAGTTGTCTCTGGTTGCAAATCTAA-3’

### HPV-16 DNA detection with quantitative PCR (qPCR) in oral rinse samples

For qPCR detection of HPV-16 DNA, specific primers were used for the amplification of a region spanning the E7 gene of the HPV-16 genome [15] (Table 1B) and primers for a housekeeping gene (β-globin, Table 1A) were run in parallel to normalize the amount of DNA input.

All samples were run in duplicate in qPCR mix containing 25–50 ng DNA, 1x iTAQ Sybr Green PCR master mix (Biorad, Hercules, CA, USA) and 0.2 μM of each primer in a total volume of 10 μL. qPCR was run on ABI Viia7 (Life Technologies, Gaithersburg, MD, USA) with the following conditions: 10 mins of denaturation at 95 °C; 40 cycles of: 95 °C (15 s), 60 °C (60s). To discriminate primer specific amplicon from primer dimers or unspecific PCR products, we also performed melt curve analysis with the following conditions: 95 °C (15 s), 60 °C (60s), 95 °C (15 s).

Standard curves were developed for HPV-16 E7 using serial dilutions of Caski-derived DNA with 66, 13.2, 2.64 and 0.528 ng DNA. Caski cells are known to have 600 viral copies per genome (6.6 pg DNA/genome) [16]. Standard curves were also developed for the housekeeping gene β-globin using the same serial dilutions of Caski DNA. This allows for the relative quantification of the input DNA level and the expression of the viral load as the number of HPV-16 E7 DNA copy number/copy of β-globin. Samples were determined as positive for HPV-16 if detection of PCR product occurred at a cycle number less than that associated with the 0 value for HPV DNA on a standard curve derived from known quantities of Caski cell HPV-16 DNA, and if the PCR product had a melt temperature of between 79 and 79.9 °C, as observed for the PCR product of control HPV-16 DNA from Caski cells.

### HPV-16 transcript detection using end-point reverse transcription PCR (RT-PCR)

Total RNA (up to 1 μg) was treated with two units of DNase I (New England Biolabs, Beverly, MA, USA) in 1x DNase Buffer (New England Biolabs) in a total volume of 10 μL to remove genomic DNA. The digestion mix was incubated at 37 °C for 10 min followed by inactivation at 75 °C for 10 min before cooling to 4 °C. To evaluate whether contaminating genomic DNA impacted on detection of HPV-16 RNA, 1 μL of selected RNA samples were subjected to cDNA synthesis without the addition of the iScript reverse transcriptase (Biorad) in a total volume of 20 μL. We then ran a PCR for GAPDH and observed no amplification products, showing that the isolated RNA was free from genomic DNA contamination after DNase treatment. DNase treated RNA was added to a cDNA synthesis reaction containing 1 μL of iScript reverse transcriptase (Biorad) and 1x iScript reaction mix in a total volume of 20 μL. The cDNA synthesis mix was incubated at 25 °C for 5 min, 42 °C for 30 min followed by enzyme inactivation at 85 °C for 5 min before cooling to 4 °C. Specific primers were used for the amplification of a region spanning the E6 gene of the HPV-16 genome and primers for a housekeeping gene (GAPDH) was run in parallel to normalize the amount of cDNA input (Table 1C). The PCR reaction mix consisted of at least 25 ng of cDNA, 1 μM of each primer, 1x EmeraldAMP MAX HS PCR mastermix (Takara Bio) in a total volume of 12.5 μL. PCR reaction condition consisted of an initial denaturation at 95 °C for 2 min followed by 40 cycles of; 95 °C for 30 s, annealing for 30 s at 58 °C, and extension at 72 °C for 30 s. A final extension at 72 °C before cooling to 4 °C was performed. The PCR products were subjected to gel electrophoresis.

### HPV-16 transcript detection using quantitative reverse transcription PCR (qRT-PCR)

Specific Taqman primers (Life Technologies) have been designed for the amplification of a region spanning HPV-16 E6 and E7 genes (Table 1D). Taqman GAPDH primers were used as endogenous control (Catalogue number 4333764 T, Life Technologies). cDNA samples were synthesized from DNAse-treated RNA as detailed above and were run in duplicate in qRT-PCR mix containing at least 25 ng cDNA, 1x Taqman Universal PCR master mix (Life Technologies) and 1x Taqman primer mix (Life Technologies). qRT-PCR was run on ABI Viia7 (Life Technologies) with the following conditions: Hold 50 °C for 2 mins; 10 mins of denaturation at 95 °C.; 40 cycles of: 95 °C (15 s), 60 °C (60s). Standard curves for the HPV-16 viral RNA copy number were developed using serial dilutions of cDNA synthesized from Caski-derived RNA. Standard curves were similarly developed for the GAPDH housekeeping gene. Viral RNA load was calculated from these standard curves as for DNA copy number, using similarly defined criteria for maximum cycle number to categorize a sample as HPV RNA positive.

### Statistical analysis

DNA and RNA copy numbers in salivary oral rinse samples were dichotomized to reflect the presence, absence nature of the data. Agreement between salivary oral rinse samples and HPV-16 status was calculated using Kappa. Sensitivity, specificity, positive, negative predictive values and Youden’s index were reported with 95 % confidence intervals. The demographic and tumor characteristics of p16^INK4a^-negative and p16^INK4a^-positive patients were examined using Fisher’s exact test (Additional file 1: Table S1) [17].

## Results

### Patient tumor characteristics

We investigated 82 patients diagnosed with HNSCC in the oral cavity, oropharynx, nasopharynx, hypopharynx, larynx, salivary gland, throat/neck and cervical lymph node (Additional file 1: Table S1). Tumor specimens from 42 of 82 (51.2 %) patients were classified as p16^INK4a^-positive and 40 (48.8 %) were p16-negative based on IHC analysis for p16^INK4a^ by qualified pathologists. p16^INK4a^-positive tumors were predominantly found in the oropharyngeal site (90.5 %), with 69 % found to be in stage IV. Whereas p16^INK4a^-negative tumors were equally spread across the three major sites (lip and oral cavity, oropharynx and larynx) with 35 % of patients in stage IV.

### HPV-16 DNA in oral rinse as a marker of tumor p16 ^INK4a^ status

**Fig. 1:**
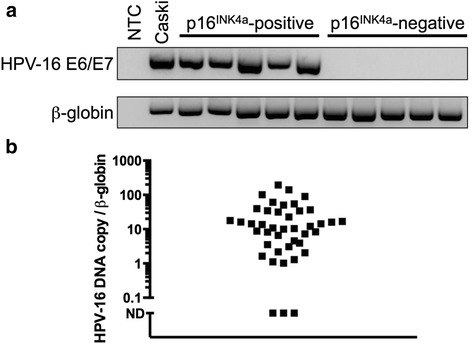
HPV-16 DNA in oral rinse associates with tumor p16^INK4a^ status. **a** Detection of HPV-16 DNA in representative oral rinse samples of patients with p16^INK4a^-positive and p16^INK4a^-negative tumors using end-point PCR. (**b**) Analysis of HPV-16 DNA copy number in oral rinse of patients with p16^INK4a^-positive tumors using quantitative RT-PCR (ND = not detected). No HPV-16 DNA was detected in oral rinse of patients with p16^INK4a^-negative tumors

We initially developed an end-point PCR-based method using primers to detect a region that spans HPV-16 E6 and E7 genome (Fig. 1a) and found high agreement between salivary oral rinse samples and p16^INK4a^ status (Kappa 0.926: 95 % CI 0.7–1.0, *p* < 0.001). 39 of the 42 patients with p16^INK4a^-positive tumors had a detectable level of HPV-16 DNA in the oral rinse samples, yielding a test sensitivity of 92.9 % and positive predictive value (PPV) of 100 % and negative predictive value (NPV) of 93 % (Table 2). No HPV-16 DNA was detected in any of the oral rinse samples of patients with p16^INK4a^-negative tumors, yielding a test specificity of predicting tumor p16 ^INK4a^ positivity of 100 % (Table 2). High agreement (Kappa 0.926: 95 % CI 0.7–1.0, *p* < 0.001) was also found using qPCR HPV-16 DNA (Fig. 1b), 39 out of 42 p16 ^INK4a^-positive patients (sensitivity = 92.9 %, PPV = 100 % and NPV = 100 %) tested positive for HPV-16 DNA in salivary oral rinse samples (Table 2) and none tested positive in the salivary oral rinse from the p16^INK4a^-negative patients (specificity = 100 %, Table 2). Additional file 2: Table S2 and Additional file 3: Table S3 summarize the results of HPV-16 detection in salivary oral fluid based on tumor p16 ^INK4a^ status.

**Table 2:** Detection of HPV-16 DNA in oral rinse samples of patients with p16-negative and p16-positive tumors

		Tumour p16^INK4a^ status			Tumour p16^INK4a^ status	
		Positive	Negative		Positive	Negative
Oral rinse HPV-16 status	Positive (Endpoint PCR)	39 (92.9 %)	0 (0.0 %)	Positive qPCR)	39 (92.9 %)	0 (0.0 %)
	Negative (Endpoint PCR)	3 (7.1 %)	40 (100.0 %)	Negative (qPCR)	3 (7.1 %)	40 (100.0 %)
	Sensitivity 0.93 (0.81, 0.99), Specificity 1.00 (0.87, 1.00) PPV 1.00 (0.87, 1.00), NPV 0.93 (0.81, 0.99) Youden 0.93 (0.68, 0.99)			Sensitivity 0.93 (0.81, 0.99), Specificity 1.00 (0.87, 1.00) PPV 1.00 (0.87, 1.00), NPV 0.93 (0.81, 0.99) Youden 0.55 (0.68, 0.99)		

### The detection of HPV-16-specific transcripts in oral fluid from patients with p16^INK4a^-positive tumors

The overexpression of HPV-16 early genes (E6 and E7) is crucial in tumor initiation and progression [17]. Therefore, PCR methods targeting HPV-specific transcripts may provide evidence of clinically relevant infection and/or persistent infection.

**Fig. 2:**
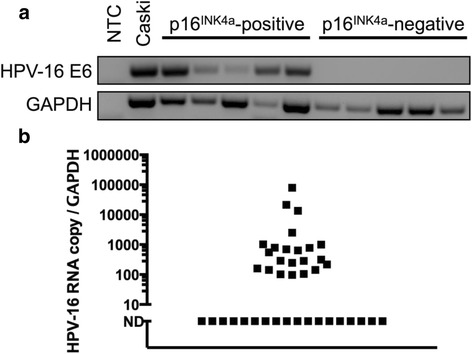
HPV-16 RNA in oral rinse associates with tumor p16^INK4a^ status. **a** Detection of HPV-16 RNA in representative oral rinse samples of patients with p16^INK4a^-positive and-negative tumors using RT-PCR. (**b**) The analysis of HPV-16 RNA copy number in oral rinse of patients with p16^INK4a^-positive tumors using quantitative RT-PCR. (ND = Not detected). No HPV-16 RNA was detected in oral rinse of patients with p16-negative tumors

We isolated RNA of sufficient quality for PCR analysis from 80 of 82 samples (see Additional file 2: Table S2 and Additional file 3: Table S3). Using reverse transcription PCR (RT-PCR), HPV-16 E6 mRNA was detected in 24 out of 40 (sensitivity = 60 %, PPV = 100 % and NPV = 71.4 %, Table 3) oral rinse samples from patients with p16 ^INK4a^-positive tumors (Fig. 2a) and no HPV-16 E6 mRNA was detected in patients with p16^INK4a^-negative tumors (specificity, 100 %, Table 3). This leads to moderate agreement between salivary oral rinse samples and HPV-16 status (Kappa 0.60: 95 % CI 0.399–0.801, *p* < 0.001).

**Table 3:** Detection of HPV-16 RNA in oral rinse samples of patients with p16-negative and p16-positive tumors

		Tumour p16^INK4a^ status			Tumour p16^INK4a^ status	
		Positive	Negative		Positive	Negative
Oral rinse HPV-16 status	Positive (Endpoint PCR)	24 (60.0 %)	0 (0.0 %)	Positive (qPCR)	22 (55.0 %)	0 (0.0 %)
	Negative (Endpoint PCR)	16 (40.0 %)	40 (100.0 %)	Negative (qPCR)	18 (45.0 %)	40 (100.0 %)
	Sensitivity 0.60 (0.43, 0.75), Specificity 1.00 (0.87, 1.00) PPV 1.00 (0.80, 1.00), NPV 0.71 (0.58, 0.83) Youden 0.6 (0.30, 0.75)			Sensitivity 0.55 (0.38, 0.71), Specificity 1.00 (0.87, 1.00) PPV 1.00 (0.78, 1.00), NPV 0.69 (0.55, 0.80) Youden 0.55 (0.26, 0.71)

Moderate agreement was also found using qRT-PCR (Kappa 0.55: 95 % CI 0.35–0.746, *p* < 0.001). HPV-16 specific E6/E7 mRNA was detected in 22 out of 40 (sensitivity = 55 %, PPV = 100 % and NPV = 69 %, Table 3) salivary oral rinse samples from p16 ^INK4a^-positive patients (Fig. 2b and Table 3) and none in the oral rinses of the 40 patients with p16^INK4a^-negative tumors (specificity = 100 %, Table 3).

## Discussion

The prevalence of HPV-positive OPSCC is rising in the western world, with more than 90 % of cases being attributed to HPV-16 infection [4]. HPV-positive OPSCC has a unique biology that is associated with improved treatment response and patient outcomes albeit an increase in recurrence [18]. The detection of primary OPSCC remains a challenge due to low accessibility of the tumor sites, which include the tonsillar crypt and base of tongue. Therefore, many OPSCC patients present with an advanced stage disease upon diagnosis [19].

Immunostaining of tumor sections for the HPV-16 surrogate marker, p16^INK4a^ is commonly used as a standalone test for the diagnosis of OPSCC. A study by Ang et al. has demonstrated that the expression of p16^INK4a^ correlated well (kappa = 0.80; 95 % CI, 0.73 to 0.87) with the presence of HPV DNA in tumors [18]. However, tumour p16^INK4a^ is an indirect marker for HPV status which is widely used by clinical pathology laboratories around the world due to its low technical costs compared to ISH and PCR based tests [20]. Therefore, there is a strong need for the correct diagnosis or detection of HPV-16 in OPSCC patients, which may enable risk stratification, patient counseling and potential de-escalation of chemo- and radio-therapies.

The sensitivity of our HPV-16 DNA test is 92.9 % in salivary oral rinse samples collected from p16^INK4a^-positive patients. This is consistent with a previous report by Koskinen et al [21] which showed a higher viral DNA load in OPSCC tumor samples compared to tumors from non-oropharyngeal sites. Using qPCR, Zhao and colleagues have found that HPV-16 DNA was detectable in 57.1 % of oral rinse samples from patients with HPV-positive tumors, with a false positive rate of 21.9 % [22]. Another study by Agrawal et al showed that only 30 % of oral rinse samples from HPV-16 positive tumors had detectable levels of HPV-16 DNA using a PCR method for the detection of L1 region of HPV DNA [23]. More recently, Ahn et al. demonstrated that the sensitivity and specificity of their saliva-based screening test using qPCR of HPV-16 E6/E7 DNA was 52.8 % for the detection of HPV-positive OPSCC in pre-treatment patients [24]. The improved sensitivity and specificity of our oral rinse-based method compared to previously published studies could be attributed to the strain specific primers used in this study. Most of the previously published work used primers that target the conserved L1 open reading frame to detect a broad spectrum of HPV strains, which contains degenerate primer sequences that may lower sensitivity [25]. Another study by Agrawal et al. showed that only 30 % of salivary oral rinse samples from HPV-16 positive tumors had detectable levels of HPV-16 DNA using a PCR method for the detection of L1 region of HPV DNA [25].

The initiation and maintenance of an HPV-driven carcinoma requires viral oncogene expression [4]. Therefore, the detection of E6/E7 mRNA transcripts has been proposed to be the ‘gold standard’ or reference test for clinically relevant infection [26]. A study by Deng et al. demonstrated that E6/E7 transcripts were detected in 15/54 (27.5 %) of the HPV-positive HNSCC tumor samples [27]. Another study by Holzinger et al. showed that E6/E7 transcripts were detected in 48/96 (50 %) OPSCC tumor samples tested positive for HPV-16 DNA [28]. To our knowledge, the current study is the first to detect HPV-16 RNA in salivary oral rinse samples (sensitivity = 60 %) as a biomarker for HPV-positive HNSCCs. Interestingly, the sensitivity of our salivary oral rinse HPV-16 RNA test was higher compared to previous findings based on tumor samples [27, 28]. However, HPV-16-specific mRNA in oral rinse has a lower sensitivity in predicting tumor p16^INK4a^ positivity compared to HPV-16 DNA. One possible reason may be that not all of the patients with HPV-16 DNA display E6/E7 mRNA expression in their tumors. Another possible cause may be due to the unstable nature of RNA in salivary oral fluids, which may have contributed to the modest test sensitivity [29]. Prior to using HPV-16 mRNA detection in routine clinical diagnosis, further work is required to optimize the protocol for the preservation of E6/E7 mRNA integrity from oral rinse samples to improve sensitivity.

We acknowledge the limitations of the current feasibility study, which include a limited sample size and results may not be generalizable to the population at large. In addition, the tumour HPV-16 status of patients was classified based on the evaluation of the surrogate marker, p16^INK4a^ instead of direct HPV-16 biomarkers such as HPV DNA or RNA. This was due to the lack of ISH or PCR analysis as part of the routine diagnostic for HPV at the Princess Alexandra Hospital, where the tumour samples were processed and analysed. Limited availability of tumour samples also hampered our ability to assess tumour HPV-16 status of the patients using direct HPV markers. However, given that up to 92.9 and 60 % of the patients with p16^INK4a^-positive tumours had detectable levels of HPV-16 DNA and RNA respectively in their oral fluid, as well as the lack of false positive in the p16^INK4a^-negative patients, strongly indicate that p16^INK4a^ positivity in tumour is associated with HPV-16 infection in the current study.

From the perspective of translation of salivary oral rinse HPV-16 assay into a routine clinical diagnostic tool, it is important to consider different origins of cells being tested in oral fluid, including tumor cells, healthy exfoliated cells and immune cells [30]. The nature of HPV infection, namely tumor-associated or an independent infection, should also be considered. Moreover, there are no standardized PCR-based methods in clinical application currently, which may lead to varied analytical sensitivities and specificities between laboratories. However, studies have shown that when used in conjunction with a standardized protocol and quality-controlled reagents, PCR-based HPV detection methods demonstrated good interlaboratory agreement [31]. Despite these limitations, our data indicate that the presence of HPV-16 DNA in oral rinse showed high agreement with HPV-related HNSCC. This is also the first study to demonstrate that HPV-16 mRNA is detectable in oral rinse of patients with HPV-related HNSCC, but the association requires further investigation.

## Conclusions

We conclude that PCR detection of HPV-16 DNA in oral rinse can serve as a diagnostic tool in HPV-16 positive HNSCC patients in addition to the current diagnostic methods. With further studies, the detection of HPV-16 in salivary oral rinse can potentially facilitate early detection of pre-cancerous lesions that may warrant further monitoring and intervention.

## Additional files

Additional file 1: Supplementary Table S1. Demographic characteristics, lifestyle and tumor characteristics of study participants (DOCX 18 kb) Additional file 2: Table S2. Summary of results for the detection of HPV-16-specific DNA and RNA in oral rinse of patients with p16^INK4a^-positive HNSCC (XLSX 41 kb) Additional file 3: Table S3. Summary of results for the detection of HPV-16-specific DNA and RNA in oral rinse of patients with p16^INK4a^-negative HNSCC (XLSX 38 kb)

## Abbreviations

ENT: ear, nose and throat
HNSCC: head and neck squamous cell carcinoma
HPV-16: human papilloma virus-16
HR-HPV: high risk-HPV
IHC: immunohistochemistry
ISH: in-situ hybridization
NPV: negative predictive value
OPSCC: oropharyngeal squamous cell carcinoma
OSCC: oral squamous cell carcinoma
PCR: polymerase chain reaction
PPV: positive predictive value
qPCR: quantitative PCR
qRT-PCR: quantitative RT-PCR
RT-PCR: reverse transcription-PCR

## References

[1] Jemal A, Bray F, Center MM, Ferlay J, Ward E, Forman D (2011). Global cancer statistics. CA Cancer J Clin.

[2] Sturgis EM, Cinciripini PM (2007). Trends in head and neck cancer incidence in relation to smoking prevalence. Cancer.

[3] Hashibe M, Brennan P, Benhamou S, Castellsague X, Chen C, Curado MP (2007). Alcohol drinking in never users of tobacco, cigarette smoking in never drinkers, and the risk of head and neck cancer: pooled analysis in the international head and neck cancer epidemiology consortium. J Natl Cancer Inst.

[4] Marur S, D'Souza G, Westra WH, Forastiere AA (2010). HPV-associated head and neck cancer: a virus-related cancer epidemic. Lancet Oncol.

[5] Chai RC, Lambie D, Verma M, Punyadeera C (2015). Current trends in the etiology and diagnosis of HPV-related head and neck cancers. Cancer Medicine.

[6] Zhang HS, Postigo AA, Dean DC (1999). Active transcriptional repression by the Rb-E2F complex mediates G1 arrest triggered by p16INK4a, TGFβ, and contact inhibition. Cell.

[7] Lima DP, Diniz DG, Moimaz SAS, Sumida DH, Okamoto AC (2010). Saliva: reflection of the body. Int J Infect Dis.

[8] Pfaffe T, Cooper-White J, Beyerlein P, Kostner K, Punyadeera C (2011). Diagnostic potential of saliva: current state and future applications. Clin Chem.

[9] Zhang X, Wan Y, Cooper-White J, Dimeski G, Atherton J, Punyadeera C (2013). Quantification of D-dimer levels in human saliva. Bioanalysis.

[10] Zhang X, Kulasinghe A, Karim R, Punyadeera C, Streckfus CF (2015). Saliva diagnostics for oral diseases. Advances in salivary diagnostics.

[11] Salazar C, Calvopiña D, Punyadeera C (2014). miRNAs in human papilloma virus associated oral and oropharyngeal squamous cell carcinomas. Expert Rev Mol Diagn.

[12] Pandit P, Cooper-White J, Punyadeera C (2013). High-yield RNA-extraction method for saliva. Clin Chem.

[13] Soler C, Chardonnet Y, Allibert P, Euvrard S, Mandrand B, Thivolet J (1992). Detection of multiple types of human papillomavirus in a giant condyloma from a grafted patient. Analysis by immunohistochemistry, in situ hybridisation, southern blot and polymerase chain reaction. Virus Res.

[14] Saiki RK, Gelfand DH, Stoffel S, Scharf SJ, Higuchi R, Horn GT (1988). Primer-directed enzymatic amplification of DNA with a thermostable DNA polymerase. Science.

[15] Walboomers JMM, Jacobs MV, Manos MM, Bosch FX, Kummer JA, Shah KV (1999). Human papillomavirus is a necessary cause of invasive cervical cancer worldwide. J Pathol.

[16] Ha PK, Pai SI, Westra WH, Gillison ML, Tong BC, Sidransky D (2002). Real-time quantitative PCR demonstrates Low prevalence of human papillomavirus type 16 in premalignant and malignant lesions of the oral cavity. Clin Cancer Res.

[17] Team RC: R: A language and environment for statistical computing. R Foundation for Statistical Computing, Vienna, Austria, 2012. In.: ISBN 3-900051-07-0; 2014. http://www.gbif.org/resource/81287.

[18] Ang KK, Harris J, Wheeler R, Weber R, Rosenthal DI, Nguyen-Tân PF (2010). Human papillomavirus and survival of patients with oropharyngeal cancer. N Engl J Med.

[19] Walvekar RR, Li RJ, Gooding WE, Gibson MK, Heron D, Johnson JT (2008). Role of surgery in limited (T1-2, N0-1) cancers of the oropharynx. Laryngoscope.

[20] Lewis J (2012). p16 immunohistochemistry as a standalone test for risk stratification in oropharyngeal squamous cell carcinoma. Head Neck Pathol.

[21] Koskinen WJ, Chen RW, Leivo I, Mäkitie A, Bäck L, Kontio R (2003). Prevalence and physical status of human papillomavirus in squamous cell carcinomas of the head and neck. Int J Cancer.

[22] Zhao M, Rosenbaum E, Carvalho AL, Koch W, Jiang W, Sidransky D (2005). Feasibility of quantitative PCR-based saliva rinse screening of HPV for head and neck cancer. Int J Cancer.

[23] Chen C-M, Shyu M-P, Au L-C, Chu H-W, Cheng WTK, Choo K-B (1994). Analysis of deletion of the integrated human papillomavirus 16 sequence in cervical cancer: a rapid multiplex polymerase chain reaction approach. J Med Virol.

[24] Ahn SM, Chan JK, Zhang Z (2014). Saliva and plasma quantitative polymerase chain reaction—based detection and surveillance of human papillomavirus—related head and neck cancer. JAMA Otolaryngology—Head & Neck Surgery.

[25] Agrawal Y, Koch WM, Xiao W, Westra WH, Trivett AL, Symer DE (2008). Oral human papillomavirus infection before and after treatment for human papillomavirus 16-positive and human papillomavirus 16-negative head and neck squamous cell carcinoma. Clin Cancer Res.

[26] Smeets SJ, Hesselink AT, Speel E-JM, Haesevoets A, Snijders PJF, Pawlita M (2007). A novel algorithm for reliable detection of human papillomavirus in paraffin embedded head and neck cancer specimen. Int J Cancer.

[27] Deng Z, Hasegawa M, Kiyuna A, Matayoshi S, Uehara T, Agena S (2013). Viral load, physical status, and E6/E7 mRNA expression of human papillomavirus in head and neck squamous cell carcinoma. Head Neck.

[28] Holzinger D, Schmitt M, Dyckhoff G, Benner A, Pawlita M, Bosch FX (2012). Viral RNA patterns and high viral load reliably define oropharynx carcinomas with active HPV16 involvement. Cancer Res.

[29] Park NJ, Li Y, Yu T, Brinkman BMN, Wong DT (2006). Characterization of RNA in saliva. Clin Chem.

[30] Venuti A, Paolini F (2012). HPV detection methods in head and neck cancer. Head Neck Pathol.

[31] Kornegay JR, Roger M, Davies PO, Shepard AP, Guerrero NA, Lloveras B (2003). International proficiency study of a consensus L1 PCR assay for the detection and typing of human papillomavirus DNA: evaluation of accuracy and intralaboratory and interlaboratory agreement. J Clin Microbiol.

