# Supplementary material for: A pilot study to compare the detection of HPV-16 biomarkers in salivary oral rinses with tumour p16INK4a expression in head and neck squamous cell carcinoma patients
Source: BMC Cancer. 2016 Mar 3;16:178. doi: 10.1186/s12885-016-2217-1 (PMC4778285; doi:10.1186/s12885-016-2217-1)
Supplement: Additional file 1: Supplementary Table S1. — Demographic characteristics, lifestyle and tumor characteristics of study participants (DOCX 18 kb) [file 12885_2016_2217_MOESM1_ESM.docx]

Supplementary Table S1.

Demographic characteristics, lifestyle and tumor characteristics of study participants

| Explanatory variables | HPV-16 negative | HPV-16 positive |  |
| --- | --- | --- | --- |
|  | n = 40 (48.8%) | n = 42 (51.2%) | P-value |
| Demographics |  |  |  |
| Gender |  |  | 0.027 |
| Male | 33 (82.5%) | 41 (97.6%) |  |
| Female | 7 (17.5%) | 1 (2.4%) |  |
| Age |  |  | 0.005 |
| <50 | 2 (5.0%) | 3 (7.1%) |  |
| 50 - 64 | 16 (40.0%) | 30 (71.4%) |  |
| >65 | 22 (55.0%) | 9 (21.4%) |  |
| Race and ethnicity |  |  | 0.024 |
| White, non-Hispanic | 35 (87.5%) | 42 (100.0%) |  |
| Other | 5 (12.5%) | 0 (0.0%) |  |
| Smoking |  |  | 0.019 |
| Non-smoker | 4 (10.0%) | 15 (35.7%) |  |
| Ex-smoker | 23 (57.5%) | 20 (47.6%) |  |
| Current smoker | 10 (25.0%) | 6(14.3%) |  |
| Unknown | 1 (2.5%) | 1 (2.4%) |  |
| Drinking* |  |  |  |
| No. of drinks per week |  |  | 0.003 |
| Non-drinker | 3 (7.5%) | 3 (7.1%) |  |
| 1 to 14 | 8 (20.0%) | 21 (50.0%) |  |
| >15 | 18 (45%) | 7 (16.7%) |  |
| Unknown | 11 (27.5%) | 11 (26.2%) |  |
| Tumour characteristics |  |  |  |
| AJCC TNM stage |  |  | 0.018 |
| Stage I | 5 (12.5%) | 1 (2.4%) |  |
| Stage II | 2 (5.0%) | 1 (2.4%) |  |
| Stage III | 7 (17.5%) | 3 (7.1%) |  |
| Stage IV | 14 (35.0%) | 29 (69.0%) |  |
| Unknown | 12 (30.0%) | 8 (19.1%) |  |
| Tumour anatomic site |  |  | <0.001 |
| Lip and oral cavity | 9 (22.5%) | 1 (2.4%) |  |
| Salivary gland | 0 (0.0%) | 1 (2.4%) |  |
| Oropharynx | 12 (30.0%) | 38 (90.5%) |  |
| Nasopharynx | 1 (2.5%) | 0 (0.0%) |  |
| Hypopharynx | 2 (5.0%) | 0 (0.0%) |  |
| Larynx | 12 (30.0%) | 1 (2.4%) |  |
| Cervical node | 1 (2.5%) | 0 (0.0%) |  |
| Throat/Neck mass | 3 (7.5%) | 1 (2.4%) |  |

* Unknown removed for analysis purposes.

Abbreviations: the American Joint Committee on Cancer for describing the extent of disease progression in cancer patients. It utilizes in part the TNM scoring system: Tumor size, Lymph Nodes affected, Metastases
